# Physical and Mental Recovery after Aortic Valve Surgery in Non-Elderly Patients: Native Valve-Preserving Surgery vs. Prosthetic Valve Replacement

**DOI:** 10.3390/jcdd10040138

**Published:** 2023-03-23

**Authors:** Theresa Holst, Johannes Petersen, Sarah Friedrich, Benjamin Waschki, Christoph Sinning, Meike Rybczynski, Hermann Reichenspurner, Evaldas Girdauskas

**Affiliations:** 1 Department of Cardiovascular Surgery, University Heart and Vascular Center Hamburg, Martinistraße 42, 20246 Hamburg, Germany; 2 Department of Cardiothoracic Surgery, Augsburg University Hospital, Stenglinstraße 2, 86156 Augsburg, Germany; 3 Department of Mathematical Statistics and Artificial Intelligence in Medicine, University of Augsburg, Universitätstraße 14, 86159 Augsburg, Germany; 4 Department of Cardiology, University Heart and Vascular Center Hamburg, Martinistraße 42, 20246 Hamburg, Germany; 5 Department of Internal Medicine, Itzehoe Hospital, Robert-Koch-Straße 2, 25524 Itzehoe, Germany

**Keywords:** aortic valve repair, Ross procedure, aortic valve replacement, quality of life, exercise capacity

## Abstract

**Background**: Exercise capacity and patient-reported outcomes are increasingly considered crucial following aortic valve (AV) surgery in non-elderly adults. We aimed to prospectively evaluate the effect of native valve preservation compared with prosthetic valve replacement. **Methods:** From October 2017 to August 2020, 100 consecutive non-elderly patients undergoing surgery for severe AV disease were included. Exercise capacity and patient-reported outcomes were evaluated upon admission, and 3 months and 1 year postoperatively. **Results:** In total, 72 patients underwent native valve-preserving procedures (AV repair or Ross procedure, NV group), and 28 patients, prosthetic valve replacement (PV group). Native valve preservation was associated with an increased risk of reoperation (weighted hazard ratio: 10.57 (95% CI: 1.24–90.01), *p* = 0.031). The estimated average treatment effect on six-minute walking distance in NV patients at 1 year was positive, but not significant (35.64 m; 95% CI: −17.03–88.30, adj. *p* = 0.554). The postoperative physical and mental quality of life was comparable in both groups. Peak oxygen consumption and work rate were better at all assessment time points in NV patients. Marked longitudinal improvements in walking distance (NV, +47 m (adj. *p* < 0.001); PV, +25 m (adj. *p* = 0.004)) and physical (NV, +7 points (adj. *p* = 0.023); PV, +10 points (adj. *p* = 0.005)) and mental quality of life (NV, +7 points (adj. *p* < 0.001); PV, +5 points (adj. *p* = 0.058)) from the preoperative period to the 1-year follow-up were observed. At 1 year, there was a tendency of more NV patients reaching reference values of walking distance. **Conclusions:** Despite the increased risk of reoperation, physical and mental performance markedly improved after native valve-preserving surgery and was comparable to that after prosthetic aortic valve replacement.

## 1. Introduction

Prosthetic aortic valve replacement (AVR) using mechanical or biological aortic valve (AV) substitutes is considered the standard of care in the treatment of non-elderly adults (i.e., age < 65 years) with AV disease, despite being associated with increased risk of anticoagulation-related thromboembolic/bleeding complications, infective endocarditis and structural valve deterioration impacting long-term survival and freedom from cardiovascular events [1,2,3,4,5].

Native valve- or living tissue-preserving procedures including AV repair and the Ross procedure are evolving alternative strategies in well-selected patients aimed at overcoming the inherent drawbacks of artificial valve substitutes and restoring survival comparable to that of the general population [6,7]. In recent decades, both procedures have become an integral part of the surgical treatment protocols in non-elderly adults presenting with severe AV disease, especially when performed at dedicated centers [8]. Both AV repair and the Ross procedure offer the potential benefit of reduced risk of valve-related complications compared with prosthetic AVR, but at the potential expense of increased risk of valve-related reoperation [6,7,9,10,11]. Moreover, both procedures potentially allow postoperative hemodynamics similar to those of well-functioning, native valves to be achieved due to the absence of a rigid sewing ring and the preservation of native aortic root geometry, permitting transvalvular flow characteristics and left ventricular dynamics to be preserved [12]. Yet, currently, no firm evidence confirming the believed superiority of native valve-preserving procedures over conventional prosthetic AVR exists, and the advantages of native valve preservation in terms of postoperative outcome determinants other than morbidity and mortality (e.g., postoperative recovery of exercise capacity as well as patient-reported outcomes) still have to be defined. Currently, only few retrospective, cross-sectional reports investigating differences in either exercise capacity [13] or patient-reported outcomes [14,15,16,17] among AV repair, Ross procedure and prosthetic AVR are available. Moreover, only two prospective studies evaluated longitudinal changes in exercise capacity alone [18] or combined with mental well-being [19] following AV surgery but without differentiating among surgical techniques. Prospective data on longitudinal changes with emphasis on the effect of the different surgical strategies (i.e., native valve-preserving procedures vs. conventional prosthetic AVR) are, however, still lacking.

The aim of this study, therefore, was to prospectively observe and evaluate the effects of living/native valve-preserving surgery (NV group) compared with prosthetic valve replacement (PV group) in non-elderly adults undergoing AV surgery as differences in potential indicators of superiority, namely, cardiopulmonary functional capacity and self-reported QoL 1 year postoperatively. Moreover, we aimed to assess any postoperative longitudinal change in physical performance and mental well-being in the cohorts (i.e., NV and PV groups).

## 2. Materials and Methods

### 2.1. Study Population

This prospective observational trial was approved by the ethics committee of General Medical Council, Hamburg, Germany (PV5723), and performed in accordance with the ethical standards as laid down in the 1964 Declaration of Helsinki and its later amendments. From October 2017 until August 2020, all patients aged 18–65 years and referred to our institution for elective AV surgery for severe isolated/predominant aortic regurgitation (AR) and non-elderly patients aged < 60 years with severe mixed congenital AV disease or severe isolated congenital aortic stenosis (AS) were considered eligible for inclusion in this study. Patients were excluded if AV dysfunction was only mild to moderate; if they suffered from isolated non-congenital aortic stenosis or syndromic congenital heart disease; if they had undergone previous cardiac surgery or intervention in childhood; or if they required concomitant mitral/tricuspid valve surgery, coronary artery bypass grafting or treatment for active endocarditis. Further exclusion criteria comprised musculoskeletal disorders or severe obesity (i.e., body weight > 150 kg) impairing mobility and thus cardiopulmonary exercise testing, or insufficient knowledge of the German language to fill out health-related questionnaires. Written informed consent was obtained from all individual subjects prior to inclusion. In total, 100 consecutive patients were prospectively included and observed during the postoperative follow-up. 

### 2.2. Surgical Procedure

Preoperative work-up included transthoracic/transesophageal echocardiography assessing the underlying mechanism of AV pathology and pulmonary valve function. A surgical attempt to preserve the living/native valvular tissue was decided together with all patients after they had been informed in detail about all three surgical options (i.e., AV repair, Ross procedure and prosthetic AVR), and their relevant benefits and drawbacks. However, the final choice of the type of AV surgery was made intraoperatively after valve exposure and detailed assessment using a standardized protocol, including the assessment of AV (and if necessary pulmonary valve) annulus diameter; the number and localization of fenestrations; the localization of calcifications; details on number of cusps and cusp fusion (i.e., right-/left-coronary, right-/non-coronary, left-/non-coronary, right-/left-coronary + right-/non-coronary, right-/left-coronary + left-/non-coronary or right-/non-coronary + left-/non-coronary, complete or partial fusion), in case of unicuspid and bicuspid AV, and commissural orientation, in case of bicuspid AV; commissural height; geometric cusp height; and effective cusp height before AV repair, Ross procedure or AVR. AV repair was performed as planned in all the patients with isolated AR in whom the tissue quality seemed sufficient for successful repair. In patients aged < 60 years with isolated/concomitant congenital AS due to a severely restrictive raphe (i.e., exclusively patients with unicuspid/bicuspid morphology), the Ross procedure was pursued as planned. In cases in whom the aortic and pulmonary valve tissue appeared unsuspectedly deficient for successful preservation upon intraoperative inspection and in cases with moderate-to-severe residual AR after a first attempt at native valve preservation, a biological or mechanical valve prosthesis was implanted according to the patient’s wish. With respect to the choice of artificial valve substitute, we deliberately advocated bioprosthetic AVR in all patients, in combination with simultaneous annulus enlargement in small aortic annuli to enable the implantation of bioprostheses with internal diameters ≥ 25 mm to be achieved to prevent postoperative patient–prosthesis mismatch and make future valve-in-valve procedures possible. If concomitant aortic root aneurysm was present, root replacement with valve reimplantation or remodeling (i.e., David or Yacoub procedure), or composite graft replacement of the AV and aortic root (i.e., Bentall procedure) was performed.

### 2.3. Study Protocol

The study protocol included the assessment of AV and left ventricular function using transthoracic echocardiography (including the quantification of AV dysfunction, and left ventricular ejection fraction and end-diastolic diameter); measures of cardiopulmonary functional capacity (including six-minute walk test (6MWT) distance [20]; cardiopulmonary exercise testing on a cycle ergometer (Vyntus CPX; Vyaire Medical, Hoechberg, Germany) using a ramp protocol and involving the estimation of peak oxygen consumption (peak VO_2_) and peak work rate); and measures of patient-reported outcomes (including the well-established 12-Item Short Form Health Survey (SF-12), evaluating self-reported physical and mental QoL with 12 items on 2 subscales [21], and Hospital Anxiety and Depression Scale (HADS), evaluating anxiety and depression with 14 items on 2 subscales [22]). All patients were assessed by a single investigator upon admission (i.e., the day before surgery) and subsequently 3 months (after having completed a cardiac rehabilitation program) and 1 year postoperatively during routine postoperative follow-up assessments at our institution. Consequently, the physical and self-reported data of each individual patient were gathered at the same time. If patients missed their follow-up appointments, they were contacted via telephone and questionnaires were subsequently mailed to them. Moreover, their referral cardiologists were contacted, and all available information (i.e., echocardiographic images and results of cardiopulmonary exercise testing) from outpatient follow-up assessments was requested for further systematic analysis. 

### 2.4. Statistical Analysis

Categorical variables are expressed as absolute and relative frequencies, and continuous variables are presented as medians (interquartile ranges) throughout the manuscript unless otherwise specified. Statistical analysis comprised three parts: (1) between-group comparison at the 1-year follow-up, and evaluation of longitudinal changes from baseline to 1-year follow-up within the (2) NV group and (3) PV group. All *p*-values were adjusted for multiple comparisons using the Bonferroni method and considered statistically significant if <0.05. In each part of the analysis, a hierarchical test procedure involving three parameters in a fixed sequence (i.e., 6MWT distance representing physical capacity → self-reported physical QoL → self-reported mental QoL) was applied. Testing was performed until the first non-rejection of the respective null hypothesis. The estimation of the average treatment effect on the treated cohort (ATT, i.e., the increase/decrease in 6MWT distance/physical QoL/mental QoL in NV patients (test group) resulting from not having required prosthetic AVR as PV patients (reference group)) at the 1-year follow-up was conducted using augmented inverse probability weighting (AIPW), a propensity score-based method involving a special form of inverse probability of treatment weighting (IPW), namely, the calculation of the so-called treated weights, with an extension to augment the estimator with a regression model for the outcome variable. This doubly robust estimator is consistent if at least one of the two models (i.e., the propensity score or the outcome model) is correctly specified. Details can be found in the published literature [23,24]. Within-group comparisons were made using the Wilcoxon sign-ranked test. Moreover, the 1-year follow-up values of patients were compared with gender- and age-specific published data on healthy individuals [21,25,26,27]. No imputation for missing values was performed. The characteristics of the remaining parameters (i.e., peak VO_2_, work rate, anxiety and depression) were summarized descriptively. Peak VO_2_ values were only included for a subset of patients (n = 65) due to a defective gas concentration sensor leading to invalid measurements until November 2018. Statistical analysis was performed until treatment failure (i.e., AV reoperation or death) as follow-up ended at that point. Time to treatment failure was analyzed using a weighted Cox regression model incorporating the ATT IPW weights. R Statistical Software (v4.0.2; R Core Team 2020), including RStudio (v1.3.1093) and the dplyr (v.1.0.7), PSW (v1.-3), survival (v3.1-12) and ggplot2 (v1.0.7) packages, was used for all statistical analyses and visualizations.

## 3. Results

### 3.1. Patient Characteristics

Baseline patient characteristics are outlined in Table 1. 

The preservation of the native valve (i.e., AV repair (n = 58) or Ross procedure (n = 14)) was possible in 72 patients, while prosthetic AVR was required in 28 patients. In total, 15/72 (21%) NV patients received valve-sparing root replacement, including 13 David procedures and 2 Yacoub procedures. In total, 2/28 (7%) PV patients received composite graft replacement of the aortic valve and root (i.e., Bentall procedure). The predominant indication for surgery was isolated AR in both groups (NV, 59/72 patients (82%); PV, 21/28 patients (75%)). The remaining patients were mostly referred for isolated AS in the NV group (10/72 patients (14%)) and for mixed AV disease in the PV group (6/28 patients (21%)). Most NV patients (78%) presented with congenital AV disease (i.e., 58% bicuspid and 19% unicuspid), while the proportions of patients with congenital AV disease (50%) and tricuspid morphology (50%) were similar in PV patients. Additionally, both groups markedly differed in terms of age, sex, sum of cardiac risk factors, severity of symptoms and overall perioperative risk profile. After IPW with the ATT as the estimand to correct for baseline differences, more patient characteristics were homogeneously distributed in both groups, as indicated by standardized mean differences ≤ 0.2 (Table 1). In Supplementary Table S1, further information on perioperative patient characteristics is summarized, including cardiopulmonary bypass duration; aortic cross-clamp time; ICU stay; and the incidence of perioperative coronary artery distortion, perioperative neurological deficit, postoperative permanent pacemaker implantation, and reintervention for complication before discharge.

### 3.2. Time to Treatment Failure

Patients were only followed up until treatment failure occurred, which was defined as either the need for AV reoperation or death. In the NV group, one patient died from small-cell lung carcinoma 8 months after surgery, and nine patients required re-do surgery for residual/recurrent severe AR during the early postoperative follow-up after initial AV repair, whereas no Ross patient required reoperation. In the PV group, one patient died from sudden cardiac death 2 months after surgery, and no patient needed re-do surgery. AV and left ventricular functional parameters in both groups determined using transthoracic echocardiography at discharge, and 3 months and 1 year postoperatively are summarized in Supplementary Table S2. Weighted Cox regression analysis incorporating the IPW-treated weights (based on parameters marked with # in Table 1) revealed increased confounder-adjusted risk of treatment failure in NV patients (weighted hazard ratio: 10.57 (95% CI: 1.24–90.01), *p* = 0.031) (Supplementary Figure S1).

### 3.3. Treatment Effect as Difference 1 Year Postoperatively

**Figure 1:**
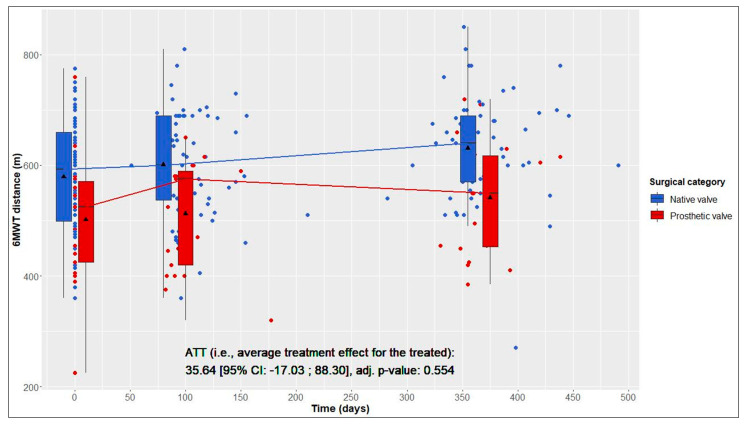
Six-minute walk test (6MWT) distance over time in native valve vs. prosthetic valve patients; boxes and whiskers indicate medians, IQRs, minima and maxima (raw data); triangles indicate means (raw data); medians are connected to show time trend; displayed *p*-value corresponds to ATT testing after inverse probability of treatment weighting.

In terms of the 6MWT 1 year postoperatively, the ATT (i.e., increase in walking distance in NV patients (test group) resulting from not having required prosthetic AVR as PV patients (reference group)) was estimated to be 35.64 m (Figure 1). 

**Figure 2:**
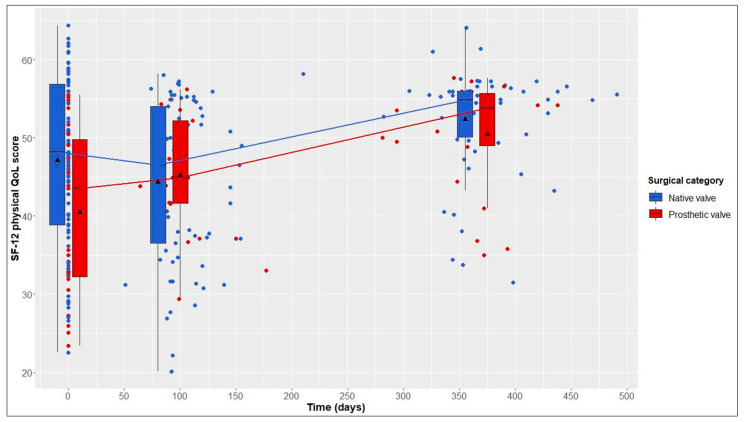
12-Item Short Form Health Survey (SF-12) physical quality of life (QoL) score over time in native valve vs. prosthetic valve patients; boxes and whiskers indicate medians, IQRs, minima and maxima (raw data); triangles indicate means (raw data); medians are connected to show time trend.

**Figure 3:**
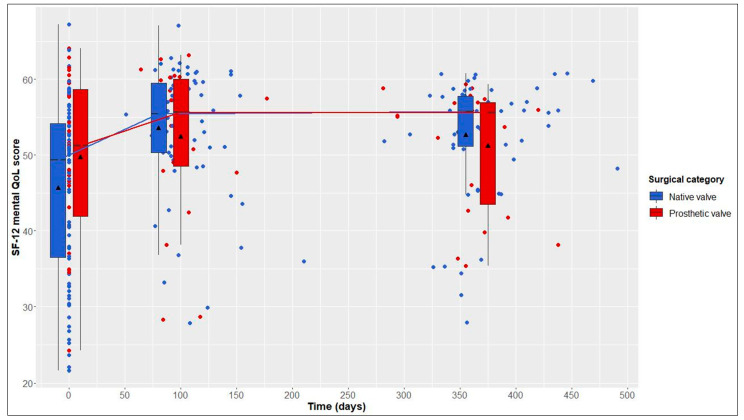
12-Item Short Form Health Survey (SF-12) mental quality of life (QoL) score over time in native valve vs. prosthetic valve patients; boxes and whiskers indicate medians, IQRs, minima and maxima (raw data); triangles indicate means (raw data); medians are connected to show time trend.

This average treatment effect on the treated group was positive, but not significant (95% CI: −17.03–88.30; adj. *p* = 0.554). According to the hierarchical test procedure, no further ATT testing was performed with respect to physical and mental QoL. According to Figure 2 and Figure 3, it can, however, be deduced that physical and mental QoL scores were similar in both groups at the 1-year follow-up (median physical QoL score, 55 (6) in NV vs. 54 (7) in PV patients; median mental QoL score, 56 (7) in NV vs. 56 (13) in PV patients).

### 3.4. Longitudinal Changes after Native Valve-Preserving Surgery

From baseline to 1-year follow-up, NV patients showed significant improvements in 6MWT distance, from 593 (161) to 640 (120) m (+47 m, adj. *p* < 0.001); in the physical QoL score, from 48 (18) to 55 (6) (+7, adj. *p* = 0.023); and in the mental QoL score, from 49 (18) to 56 (7) (+7, adj. *p* < 0.001) (Table 2 and Figure 1, Figure 2 and Figure 3).

### 3.5. Longitudinal Changes after Prosthetic Valve Replacement

From baseline to 1-year follow-up, PV patients also showed significant improvements in 6MWT distance, from 525 (146) to 550 (165) m (+25 m, adj. *p* = 0.004), and in the physical QoL score, from 44 (18) to 54 (7) (+10, adj. *p* = 0.005). Although not statistically significant, improvements in the mental QoL score were also observed (baseline, 51 (17), vs. 1 year postoperatively, 56 (13); +5, adj. *p* = 0.058) (Table 2 and Figure 1, Figure 2 and Figure 3).

### 3.6. Differences between Our Data and Gender- and Age-Specific Published Data on Healthy Individuals

**Figure 4:**
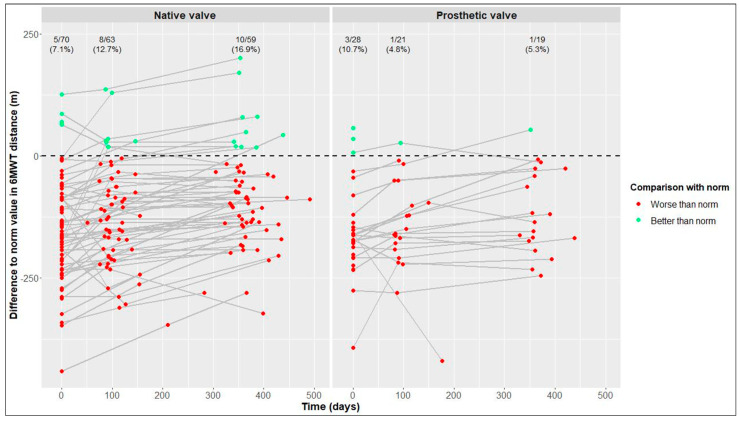
Differences between our values and reference values of six-minute walk test (6MWT) distance [25] in native valve vs. prosthetic valve patients; measurements of individual patients are represented by separate dots and connected to show time trend; absolute and relative frequencies of patients reaching reference values are displayed (raw data).

One year postoperatively, the percentages of patients reaching reference values of the 6MWT derived from published data on healthy individuals were 16.9% of the NV group and only 5.3% of the PV group (Figure 4). 

**Figure 5:**
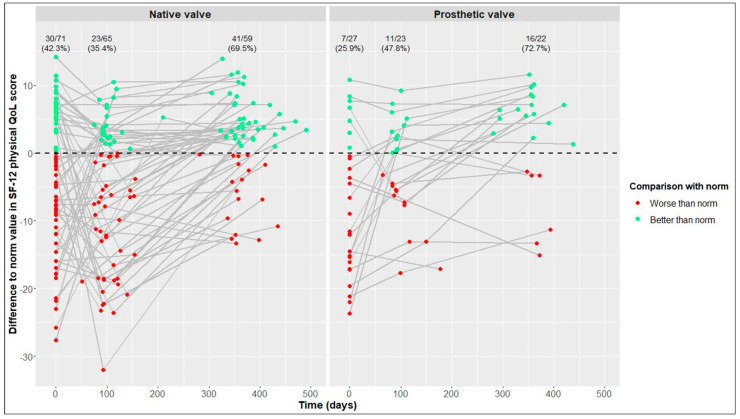
Differences between our values and reference values of 12-Item Short Form Health Survey (SF-12) physical quality of life (QoL) [21] in native valve vs. prosthetic valve patients; measurements of individual patients are represented by separate dots and connected to show time trend; absolute and relative frequencies of patients reaching reference values are displayed (raw data).

**Figure 6:**
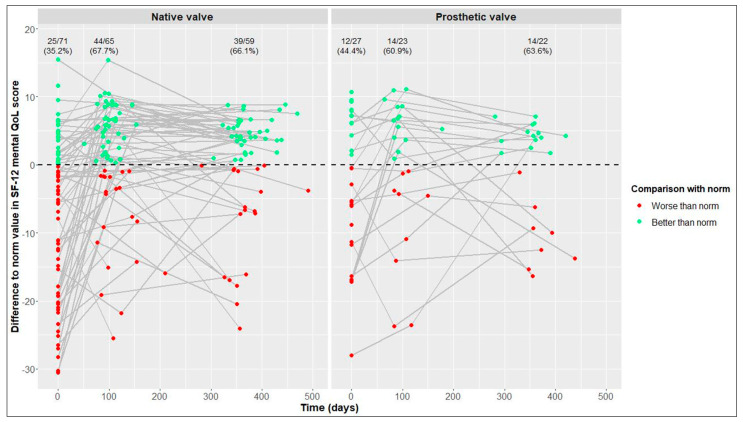
Differences between our values and reference values of 12-Item Short Form Health Survey (SF-12) mental quality of life (QoL) [21] in native valve vs. prosthetic valve patients; measurements of individual patients are represented by separate dots and connected to show time trend; absolute and relative frequencies of patients reaching reference values are displayed (raw data).

In terms of physical and mental QoL (Figure 5 and Figure 6), the percentages of patients reaching reference values at the 1-year follow-up were similar in the NV and PV cohorts (physical QoL, 69.5% vs. 72.7%, respectively; mental QoL, 66.1% vs. 63.6%, respectively).

### 3.7. Descriptive Statistics of Peak VO_2_, Work Rate, Anxiety and Depression

From baseline to 1 year postoperatively, improvements in median peak VO_2_ and work rate were +3.7 mL/kg/min and +33.5 Watts in NV patients vs. +1.35 mL/kg/min and +13.5 Watts in PV patients. During the same period, median anxiety and depression scores decreased by 3 and 3 points in NV patients vs. 2 and 2.5 points in PV patients, respectively. At all times, including baseline assessment, median peak VO_2_ and peak work rate were better in NV patients than in PV patients, while median anxiety and depression scores were similar in both groups (Supplementary Figures S2–S9).

## 4. Discussion

Despite progress in artificial AV substitutes in terms of design and function over time and the evolution of new therapeutic strategies attempting native valve preservation, the treatment of non-elderly adults with AV disease remains a challenge due to the unique characteristics of this otherwise relatively healthy patient cohort: (1) longer anticipated life expectancy imposing higher cumulative risk of valve-related complications; (2) higher levels of physical and metabolic activity; and (3) a major focus on patient-reported outcomes with greater importance of preserved or restored physical, mental and social functioning [28]. When evaluating different treatment strategies in these patients, it is thus crucial to also focus on physical capacity and patient-reported outcomes besides morbidity and mortality. 

In our patients, native valve preservation was associated with increased risk of treatment failure, more specifically, AV reoperation for failed AV repair, in NV patients, which is in line with previous findings [11,29,30]. A learning curve with a more liberal/aggressive approach to AV repair at the beginning of the study (i.e., performing AV repair in unicuspid morphology using the bicuspidization procedure [31], in bicuspid morphology with large calcifications and a severely restrictive raphe, and in bicuspid/tricuspid morphology with large fenestrations necessitating patch augmentation) might have contributed to this. Patients were only followed up until treatment failure, and the statistical analysis needs to be interpreted accordingly. 

The benefit of the preservation of native valve tissue, avoiding prosthetic AVR and its inherent drawbacks, including the concurring lifetime risk of either anticoagulation-related complications or structural valve deterioration [1,2,3,4], translated into a positive estimated average treatment effect of 35.64 m with respect to 6MWT distance in NV patients compared with PV patients. This positive average treatment effect in NV patients was, however, not significant. The subsequent descriptive statistical analysis of physical and mental QoL showed similar scores at the 1-year follow-up in both groups. This is in agreement with a recent study that reported no significant differences in physical and mental QoL in children and young adults following the Ross procedure vs. mechanical AVR [16], but contrary to most previous reports evaluating and comparing QoL in adult patients undergoing AV repair, the Ross procedure and mechanical AVR, Nötzold and colleagues observed that postoperative physical and mental QoL is quite influenced by the type of AV procedure and negatively linked with mechanical AVR compared with the Ross procedure [17]. These findings were later confirmed by two other groups and extended from Ross to AV repair patients [14,15]. The fact that 86% of our AVR patients had received a biological instead of mechanical valve substitute might have accounted for our results being inconsistent with those previous investigations. 

Yet, we still confirmed the previous findings obtained by Aicher and colleagues with respect to HADS subscales by also observing similar anxiety and depression levels 1 year after native valve-preserving surgery and prosthetic AVR [14].

Only peak VO_2_ and work rate were considerably better in NV patients than in PV patients 1 year postoperatively, which is in agreement with a recently published study that reported better initial postoperative exercise capacity assessed with cardiopulmonary exercise testing in children and young adults after the Ross procedure than after mechanical AVR at the mid-term follow-up [13]. However, it must be pointed out that the observed between-group differences in our patients were already present at baseline and were potentially linked to differences in age and sex distribution in both groups. 

Physical and mental recovery in terms of notable longitudinal improvements in 6MWT distance and self-reported physical and mental QoL during the first postoperative year was observed following both native valve preservation and prosthetic AVR. Considerable decreases in self-reported anxiety and depression from baseline to 1-year follow-up were also seen in both cohorts. Our findings are in line with previous work by Petersen and colleagues [19], who assessed the course of physical and mental recovery after AV surgery during the first 6 months postoperatively using the same instruments, but without differentiating among different surgical techniques. The observed improvements in physical and mental QoL, as well as in anxiety and depression, in their patients were similar to ours, while the improvement in 6MWT distance in their patients was markedly greater. This is likely the result of both our patient cohorts already performing better in the 6MWT (i.e., walking faster and consequently further) at baseline, thus leaving less room for improvement during follow-up, as subjects are only allowed to walk and are not allowed to run, even if possible [20]. Additionally, the better baseline performance might reflect the impact of earlier indication for AV surgery in asymptomatic patients as recommended by the recent ESC/EACTS guidelines for the management of valvular heart disease [32].

To our knowledge, cardiopulmonary exercise testing has not yet been investigated in great detail following AV repair or the Ross procedure specifically. We found small improvements in median peak VO_2_ and work rate at 1-year follow-up in NV and PV patients. The improvements were, however, slightly more pronounced following native valve preservation than after prosthetic AVR and were also more pronounced compared with previous findings by Tamás and colleagues, who performed cardiopulmonary exercise testing before AV surgery and 6 months after AV surgery and observed steady peak VO_2_ and an increase of only 12 Watts in peak work rate postoperatively. Their study cohort included mostly prosthetic AVR patients and only three patients with reconstructive surgery [18]. 

In previous works, average values of study cohorts were compared with reference values derived from published data on healthy individuals [14,19]. In contrast, we calculated, for each patient, the difference between his/her value and his/her gender- and age-specific reference values at baseline and follow-up and then determined the absolute/relative frequency of patients reaching reference values for each visit. One year postoperatively, there was a tendency of more NV patients than PV patients reaching the reference values of the 6MWT, while the percentages of patients reaching the reference values of physical and mental QoL were similar in both cohorts.

### Limitations

As we report a single-center experience, the generalization of our findings is limited. Patients were not actively assigned to a group, and sample sizes were not determined in advance but rather resulted from the impossibility to preserve the living/native valvular tissue in some patients as established intraoperatively, which led to uneven sample sizes. Moreover, we only present short-term effects due to the limited follow-up period of 1 year. To derive conclusions on mid- and long-term benefits and risks of native valve preservation compared with prosthetic AVR, a longer follow-up is required. Furthermore, follow-up ended at the time of treatment failure, so the impact of AV reoperation on physical capacity and mental well-being could not be determined. Hence, the conclusions drawn are technically only valid until a patient requires reoperation, which might introduce a slightly biased, positive view for the NV group. The ATT estimated using AIPW should also be interpreted with caution, as the unmeasured confounders, heterogenous treatment groups and poor small sample size properties of causal inference methods might have hindered the correct specification of either the propensity score or the outcome model; therefore, results might again be slightly biased. In fact, even after IPW with the calculation of the treated weights, some patient characteristics, including the presence of two or more cardiac risk factors and the underlying AV pathology, remained heterogeneously distributed in both groups, as indicated by standardized mean differences > 0.2. In addition, the cardiopulmonary exercise testing parameters were only summarized descriptively, as peak VO_2_ values were solely available for a subset of patients due to invalid measurements until November 2018. In summary, our findings should be confirmed in further prospective, ideally multicentric studies with larger sample sizes and more homogenous patient cohorts. A valve-specific questionnaire should be added to obtain more specific insights into QoL after valve surgery. Furthermore, follow-up should be continued after AV reoperation to enable results to be interpretated independently of the risk of treatment failure.

## 5. Conclusions

- Physical and mental performance improved during the first year after native valve preservation and prosthetic AVR.
- One year postoperatively, the reported physical and mental QoL was similar in both cohorts, while native valve preservation was associated with a positive, although not significant, treatment effect on 6MWT distance.
- A tendency of more patients reaching the 6MWT distance of healthy individuals and a trend of better peak oxygen consumption and work rate at the 1-year follow-up following native valve-preserving surgery than following prosthetic AVR were observed.
- Despite an increased risk of treatment failure, physical and mental performance after native valve-preserving surgery was comparable to that after conventional prosthetic AVR.
- Hence, shared decision making with patients to choose the appropriate treatment option adapted to their own specific needs is necessary.

### Contributions to the Field

Despite its limitations, our study is the first study to provide prospective data on early longitudinal postoperative changes in both physical and mental capacity after AV surgery in non-elderly patients with an emphasis on the effect of modern living/native valve-preserving procedures compared with conventional prosthetic AVR. It underlines the value of AV repair and the Ross procedure in today’s surgical armamentarium for treating AV disease in non-elderly patients by recognizing it as a reasonable alternative to prosthetic AVR, which is still considered the standard of care in most centers in spite of its inherent long-term risks and drawbacks. Patients should, therefore, have sufficient information and a sufficient understanding of the existence, as well as associated risks and benefits, of native valve-preserving procedures before making a decision about the treatment of their AV disease.

## Figures and Tables

**Table 1:** Baseline patient characteristics.

	NV (n = 72)	PV before Weighting (n = 28)	PV after Weighting (Weight: 76.92)	SMD before Weighting	SMD after Weighting
Age (years) ^#$^	41 ± 12	52 ± 12	40 ± 13	0.92	0.10
Male sex ^#$^	62 (86%)	20 (71%)	68 (88%)	0.37	0.07
Sum of cardiac risk factors *^#$^					
0	9 (13%)	2 (7%)	11 (14%)	0.20	0.04
1	20 (28%)	5 (18%)	15 (20%)	0.24	0.18
2	30 (42%)	9 (32%)	16 (21%)	0.21	0.47
3	9 (13%)	8 (29%)	18 (24%)	0.40	0.29
≥4	4 (6%)	4 (14%)	17 (22%)	0.27	0.54
AV morphology ^#^					
Unicuspid	14 (19%)	4 (14%)	15 (20%)	0.14	0.00
Bicuspid	42 (58%)	10 (36%)	49 (64%)	0.45	0.11
Tricuspid	16 (22%)	14 (50%)	13 (17%)	0.61	0.14
Reason for surgery ^$^					
Isolated regurgitation	59 (82%)	21 (75%)	54 (70%)	0.17	0.28
Isolated stenosis	10 (14%)	1 (4%)	1 (1%)	0.35	0.50
Mixed AV disease	3 (4%)	6 (21%)	22 (29%)	0.53	0.70
NYHA class ^#$^					
I	33 (46%)	8 (29%)	37 (48%)	0.36	0.03
II	28 (39%)	9 (32%)	34 (44%)	0.15	0.10
III	11 (15%)	11 (39%)	7 (9%)	0.56	0.21
IV	0 (0%)	0 (0%)	0 (0%)	0.00	0.00
Preoperative proBNP (ng/L) ^#$^	332 ± 634	1102 ± 1882	372 ± 594	0.55	0.07
Preoperative LVEF (%) ^#$^	56 ± 7	52 ± 8	55 ± 8	0.53	0.04
Preoperative LVESD_ind_ (mm/m^2^)	21 ± 3	24 ± 6	22 ± 2	0.63	0.10
STS-PROM (%) ^#$^	0.75 ± 0.59	0.92 ± 0.51	0.82 ± 0.37	0.31	0.14
EuroSCORE II (%) ^#$^	1.19 ± 0.99	1.20 ± 0.65	1.29 ± 0.54	0.11	0.12

Data presented as means ± SD or absolute and relative frequencies. * includes hypertension, hyperlipidemia, diabetes, BMI, obesity, smoking, creatinine level, coronary artery disease, extracardiac arteriopathy, previous stroke and previous cardiac surgery in adulthood. ^#^ parameters included in propensity score model. ^$^ parameters included in outcome model. AV, aortic valve; LVEF, left ventricular ejection fraction; LVESD, left ventricular end-systolic diameter; NV, native valve; PROM, predicted risk of mortality; PV, prosthetic valve; proBNP, brain natriuretic peptide; SMD, standardized mean difference (i.e., difference in means divided by the standard deviation).

**Table 2:** Longitudinal changes from baseline to 1-year follow-up.

		Baseline	One-Year Follow-Up	Adj. *p*-Value *
6MWT distance	Native valve	593 (161)	640 (120)	<0.001
	Prosthetic valve	525 (146)	550 (165)	0.004
SF-12 physical QoL	Native valve	48 (18)	55 (6)	0.023
	Prosthetic valve	44 (18)	54 (7)	0.005
SF-12 mental QoL	Native valve	49 (18)	56 (7)	<0.001
	Prosthetic valve	51 (17)	56 (13)	0.058

Data presented as medians (IQRs). * derived from Wilcoxon sign-ranked test. 6MWT, six-minute walk test; QoL, quality of life; SF-12, 12-Item Short Form Health Survey.

## Data Availability

The data underlying this article will be shared upon reasonable request to the corresponding author.

## Supplementary Materials

The following supporting information can be downloaded at: https://www.mdpi.com/article/10.3390/jcdd10040138/s1, Figure S1: Plot of confounder-adjusted time to treatment failure (i.e., aortic valve reoperation or death) in native valve patients vs. prosthetic valve patients based on Cox regression analysis incorporating the inverse probability of treatment weights. Confounders: parameters included in the propensity score model marked with # in Table 1 of the main text, Figure S2: Peak oxygen consumption (peak VO_2_) assessed with cardiopulmonary exercise testing over time in native valve patients vs. prosthetic valve patients; boxes and whiskers indicate medians, IQRs, minima and maxima (raw data); triangles indicate means (raw data); medians are connected to show time trend, Figure S3: Work rate at peak oxygen consumption assessed with cardiopulmonary exercise testing over time in native valve patients vs. prosthetic valve patients; boxes and whiskers indicate medians, IQRs, minima and maxima (raw data); triangles indicate means (raw data); medians are connected to show time trend, Figure S4: Self-reported anxiety on Hospital Anxiety and Depression Scale (HADS) over time in native valve patients vs. prosthetic valve patients; boxes and whiskers indicate medians, IQRs, minima and maxima (raw data); triangles indicate means (raw data); medians are connected to show time trend, Figure S5: Self-reported depression on Hospital Anxiety and Depression Scale (HADS) over time in native valve patients vs. prosthetic valve patients; boxes and whiskers indicate medians, IQRs, minima and maxima (raw data); triangles indicate means (raw data); medians are connected to show time trend, Figure S6: Differences between our values and reference values of peak oxygen consumption (peak VO_2_) assessed with cardiopulmonary exercise testing [26] in native valve patients vs. prosthetic valve patients; measurements of individual patients are represented by separate dots and connected to show time trend; absolute and relative frequencies of patients reaching reference values are displayed (raw data), Figure S7: Differences between our values and reference values of work rate at peak oxygen consumption assessed with cardiopulmonary exercise testing [26] in native valve patients vs. prosthetic valve patients; measurements of individual patients are represented by separate dots and connected to show time trend; absolute and relative frequencies of patients reaching reference values are displayed (raw data), Figure S8: Differences between our values and reference values of self-reported anxiety on Hospital Anxiety and Depression Scale (HADS) [27] in native valve patients vs. prosthetic valve patients; measurements of individual patients are represented by separate dots and connected to show time trend; absolute and relative frequencies of patients reaching reference values are displayed (raw data), Figure S9: Differences between our values and reference values of self-reported depression on Hospital Anxiety and Depression Scale (HADS) [27] in native valve patients vs. prosthetic valve patients; measurements of individual patients are represented by separate dots and connected to show time trend; absolute and relative frequencies of patients reaching reference values are displayed (raw data), Table S1: Perioperative patient characteristics, Table S2: Echocardiographic parameters at discharge, and 3 months and 1 year postoperatively.
