# Supplementary material for: Physical and Mental Recovery after Aortic Valve Surgery in Non-Elderly Patients: Native Valve-Preserving Surgery vs. Prosthetic Valve Replacement
Source: J Cardiovasc Dev Dis. 2023 Mar 23;10(4):138. doi: 10.3390/jcdd10040138 (PMC10146276; doi:10.3390/jcdd10040138)
Supplement: Supplementary file 1 [file jcdd-10-00138-s001.zip › jcdd-2258350_Supplementary_Material update.pdf]

Supplementary Materials

Physical and Mental Recovery after Aortic Valve Surgery in Non-Elderly Patients: Native Valve-Preserving Surgery vs. Prosthetic Valve Replacement

Theresa Holst [1,2,*,†], Johannes Petersen [1,†], Sarah Friedrich [3], Benjamin Waschki [4,5], Christoph Sinning [4], Meike Rybczynski [4], Hermann Reichenspurner [1] and Evaldas Girdauskas [1,2,*]

* Correspondence: theresa.holst@uk-augsburg.de (T.H.); evaldas.girdauskas@uk-augsburg.de (E.G.)

1 Supplementary Tables

Table S1. Perioperative patient characteristics.

	**NV (n=72)**	**PV (n=28)**
Cardiopulmonary bypass duration (min)	139±54	123±45
Aortic cross-clamp time (min)	90±43	82±29
Intensive care unit stay (days)	1.6±1.2	2.2±1.6
Perioperative coronary artery distortion	2 (3%)	0 (0%)
Perioperative neurological deficit	0 (0%)	1 (4%)
Postoperative pacemaker implantation	4 (6%)	4 (14%)
Reintervention for complication before discharge	6 (8%)	3 (11%)

Data presented as means ± SD or absolute and relative frequencies. Raw data presented.

NV: native valve; PV: prosthetic valve

Table S2. Echocardiographic parameters at discharge and 3 months and 1 year postoperatively.

	**NV (n=72)**	**PV (n=28)**
Residual AR at discharge<br>- *no/trace*<br>- *mild*<br>- *moderate*<br>- *severe*	 44 (61%)<br>23 (34%)<br>1 (2%)<br>0 (0%)	 26 (93%)<br>2 (7%)<br>0 (0%)<br>0 (0%)
Max. AV gradient at discharge (mmHg)	17±10	18±8
Mean AV gradient at discharge (mmHg)	9±6	10±4
LVEF at discharge (%)	49±7	46±11
$LVESD_{ind}$ at discharge (mm/m^2)	21±3	23±5
Residual AR at 3 months<br>- *no/trace*<br>- *mild*<br>- *moderate*<br>- *severe*	 25 (37%)<br>32 (48%)<br>7 (10%)<br>3 (4%)	 22 (88%)<br>3 (12%)<br>0 (0%)<br>0 (0%)
Max. AV gradient at 3 months (mmHg)	15±7	16±5
Mean AV gradient at 3 months (mmHg)	8±4	9±2
LVEF at 3 months (%)	54±7	56±10
$LVESD_{ind}$ at 3 months (mm/m^2)	19±3	20±4
Residual AR at 1 year<br>- *no/trace*	 20 (33%)	 22 (92%)

- *mild*<br>- *moderate*<br>- *severe*	34 (56%)<br>7 (12%)<br>0 (0%)	2 (8%)<br>0 (0%)<br>0 (0%)
Max. AV gradient at 1 year (mmHg)	13±8	17±7
Mean AV gradient at 1 year (mmHg)	7±5	9±3
LVEF at 1 year (%)	54±7	55±5
$LVESD_{ind}$ at 1 year (mm/m^2)	19±3	19±3

Data presented as means ± SD or absolute and relative frequencies. Raw data presented.

AV: aortic valve; AR: aortic regurgitation; LVESD: left ventricular end-systolic diameter; LVEF: left ventricular ejection fraction; NV: native valve; PV: prosthetic valve

2 Supplementary Figures

Figure S1. Plot of confounder-adjusted time to treatment failure (i.e., aortic valve reoperation or death) in native valve patients vs. prosthetic valve patients based on Cox regression analysis incorporating the inverse probability of treatment weights (treated weights); Confounders: parameters included in the propensity score model marked with # in **Table 1** of the main text.

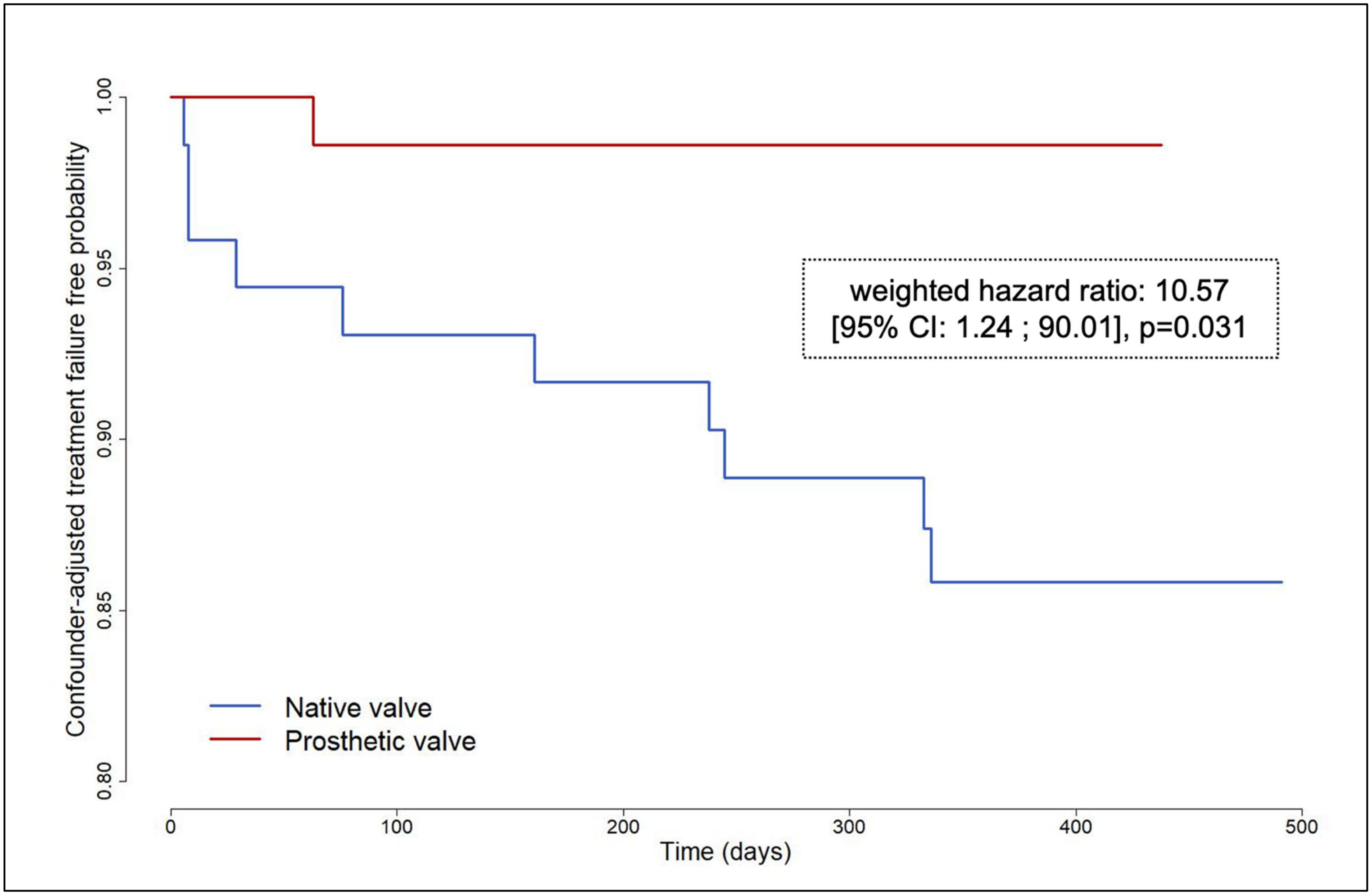

Figure S2. Peak oxygen consumption (peak VO_2) assessed with cardiopulmonary exercise testing over time in native valve patients vs. prosthetic valve patients; boxes and whiskers indicate median, IQR, minima and maxima (raw data); triangles indicate means (raw data); medians are connected to show time trend.

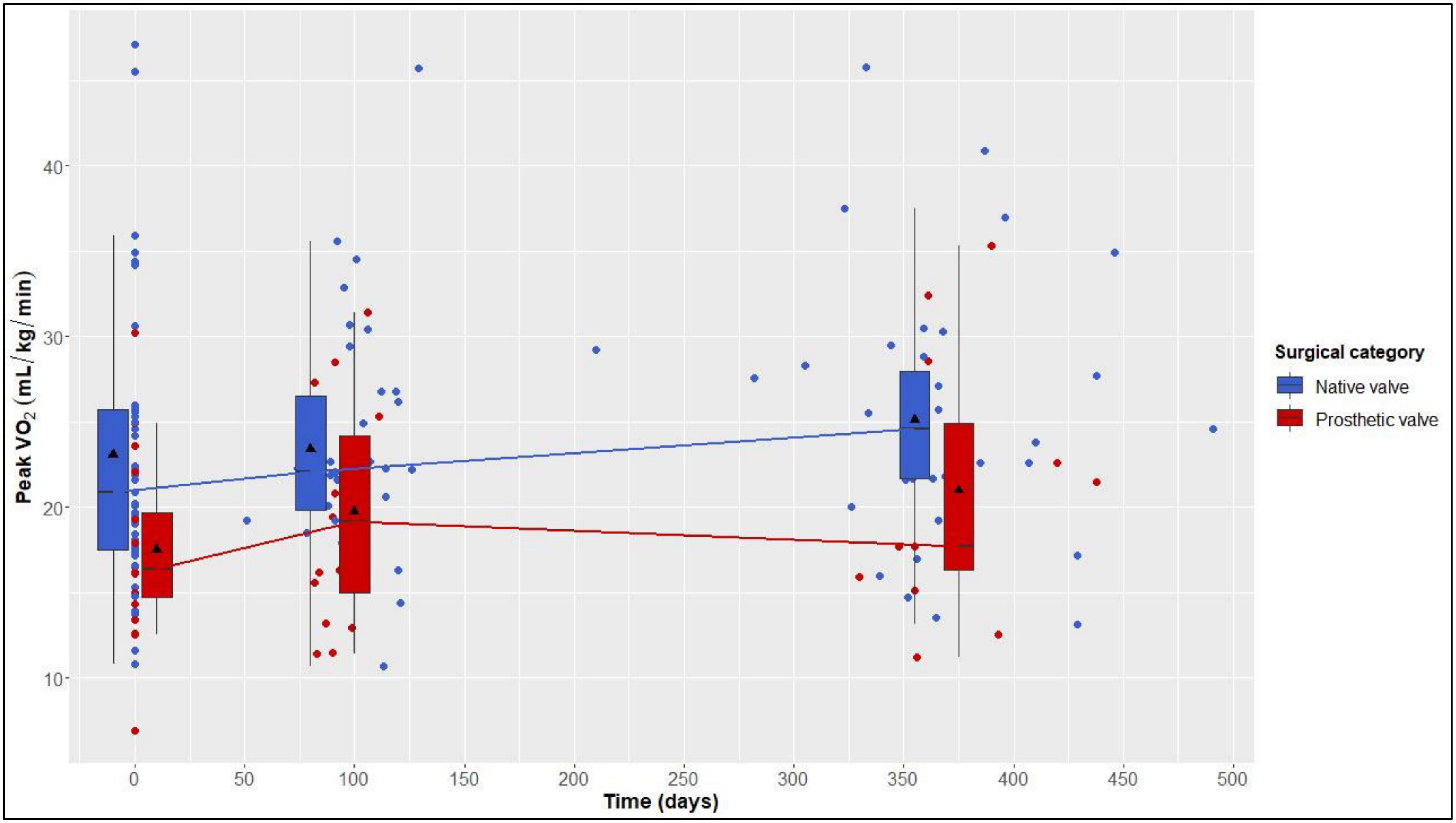

Figure S3. Work rate at peak oxygen consumption assessed with cardiopulmonary exercise testing over time in native valve patients vs. prosthetic valve patients; boxes and whiskers indicate median, IQR, minima and maxima (raw data); triangles indicate means (raw data); medians are connected to show time trend.

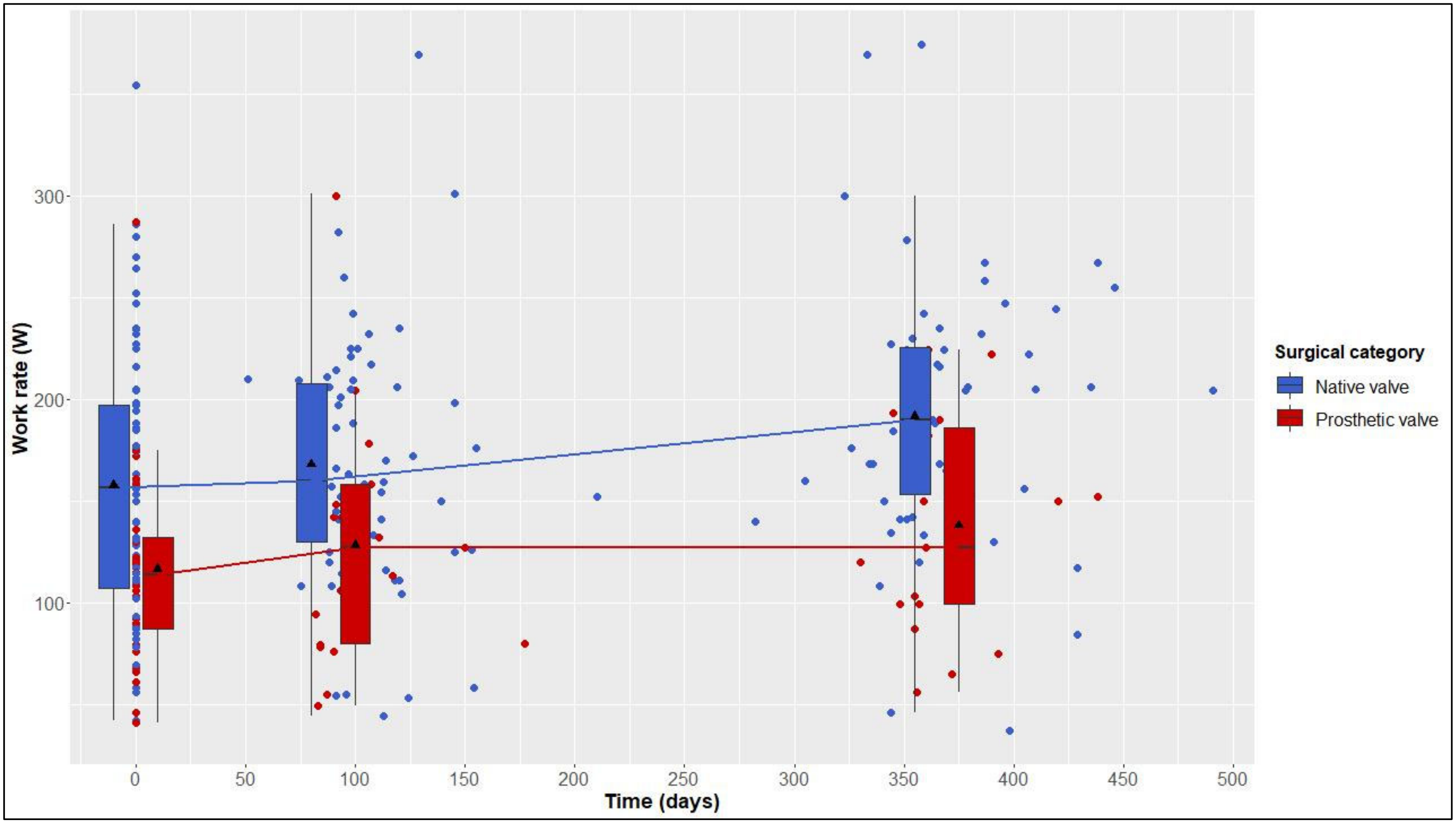

Figure S4. Self-reported anxiety on Hospital Anxiety and Depression Scale (HADS) over time in native valve patients vs. prosthetic valve patients; boxes and whiskers indicate median, IQR, minima and maxima (raw data); triangles indicate means (raw data); medians are connected to show time trend.

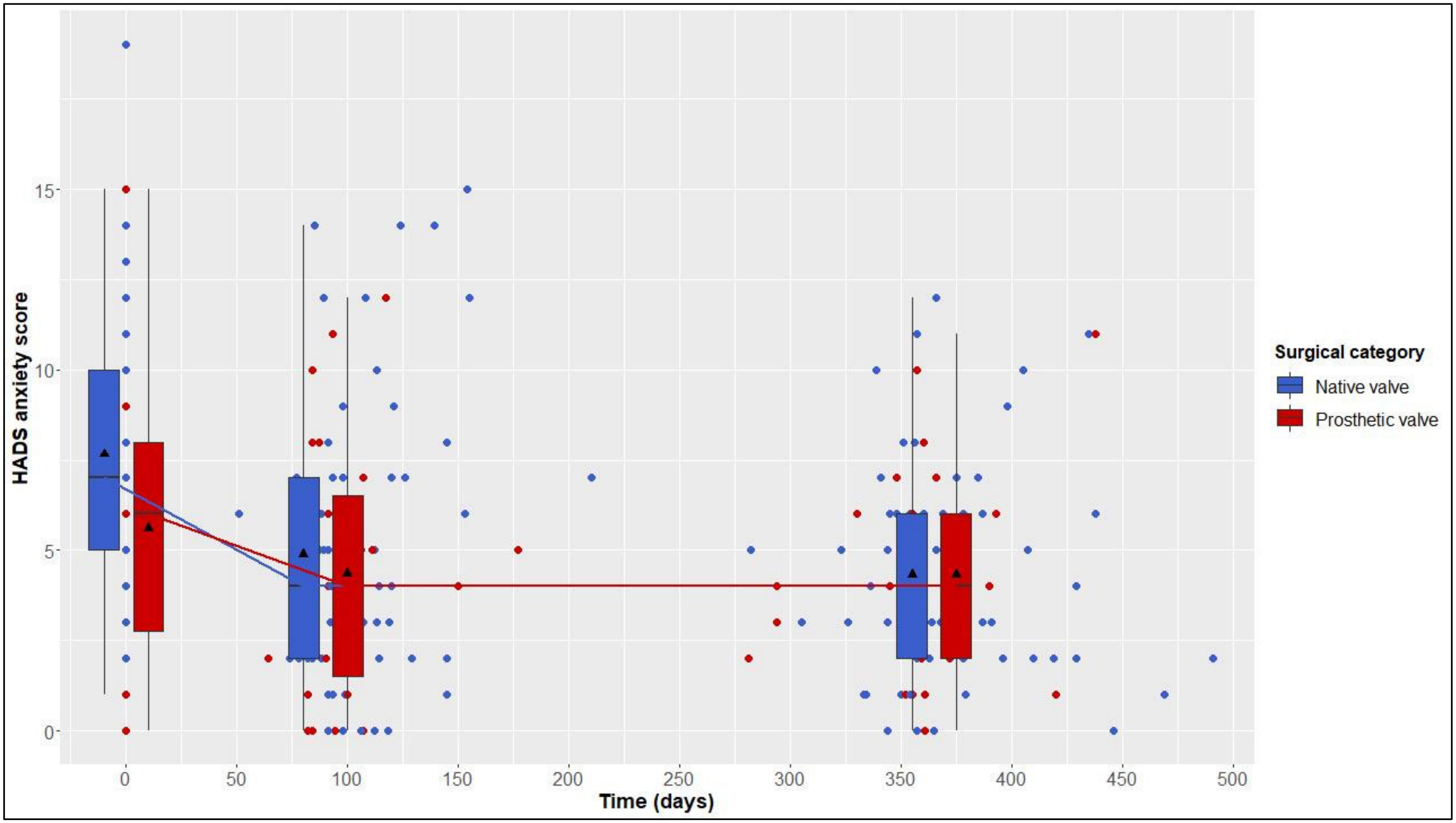

Figure S5. Self-reported depression on Hospital Anxiety and Depression Scale (HADS) over time in native valve patients vs. prosthetic valve patients; boxes and whiskers indicate median, IQR, minima and maxima (raw data); triangles indicate means (raw data); medians are connected to show time trend.

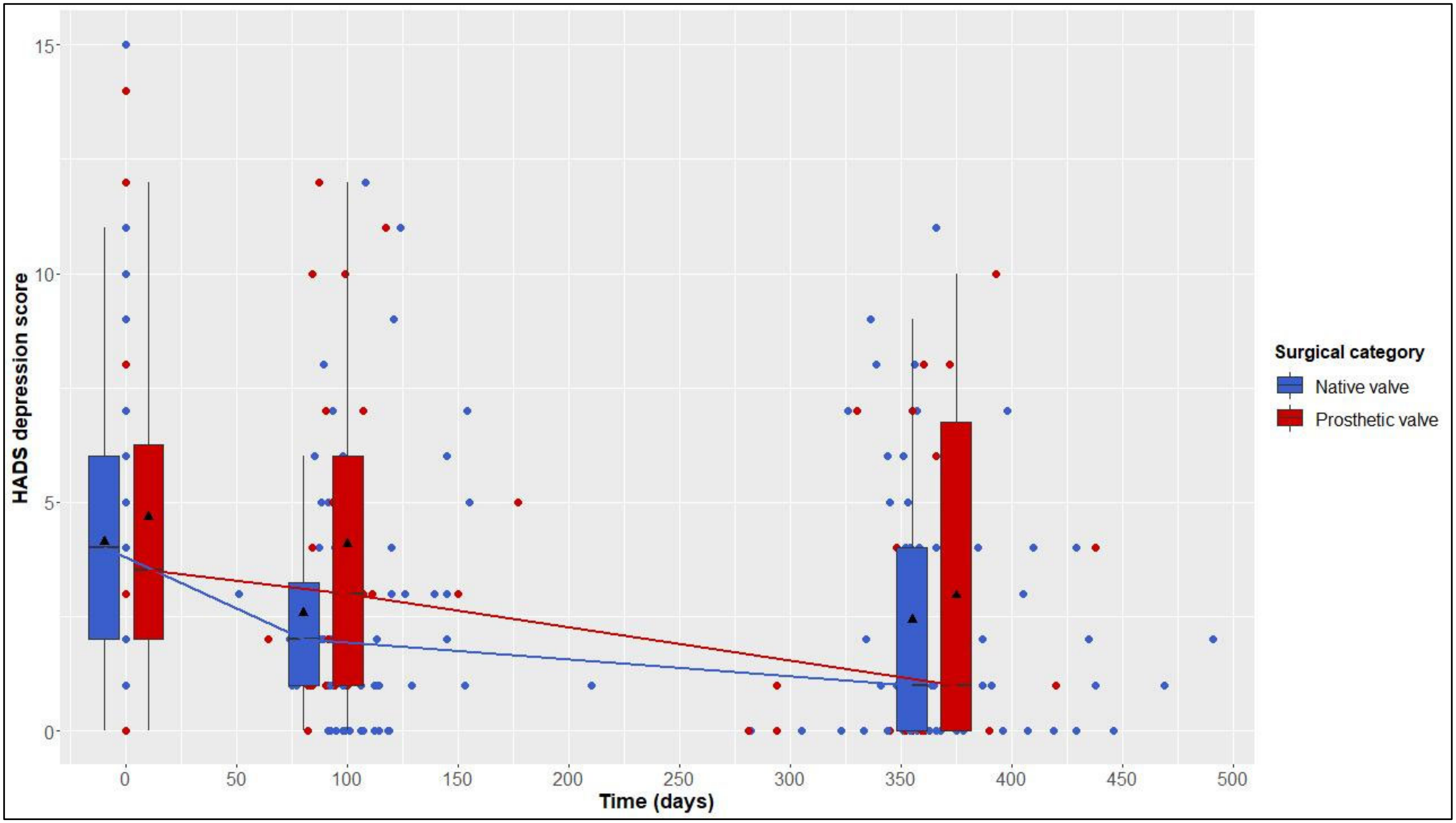

Figure S6. Difference between our values and reference values of peak oxygen consumption (peak VO_2) assessed with cardiopulmonary exercise testing (1) in native valve patients vs. prosthetic valve patients; measurements of individual patients are represented by separate dots and connected to show time trend; absolute and relative frequencies of patients reaching reference values are displayed (raw data).

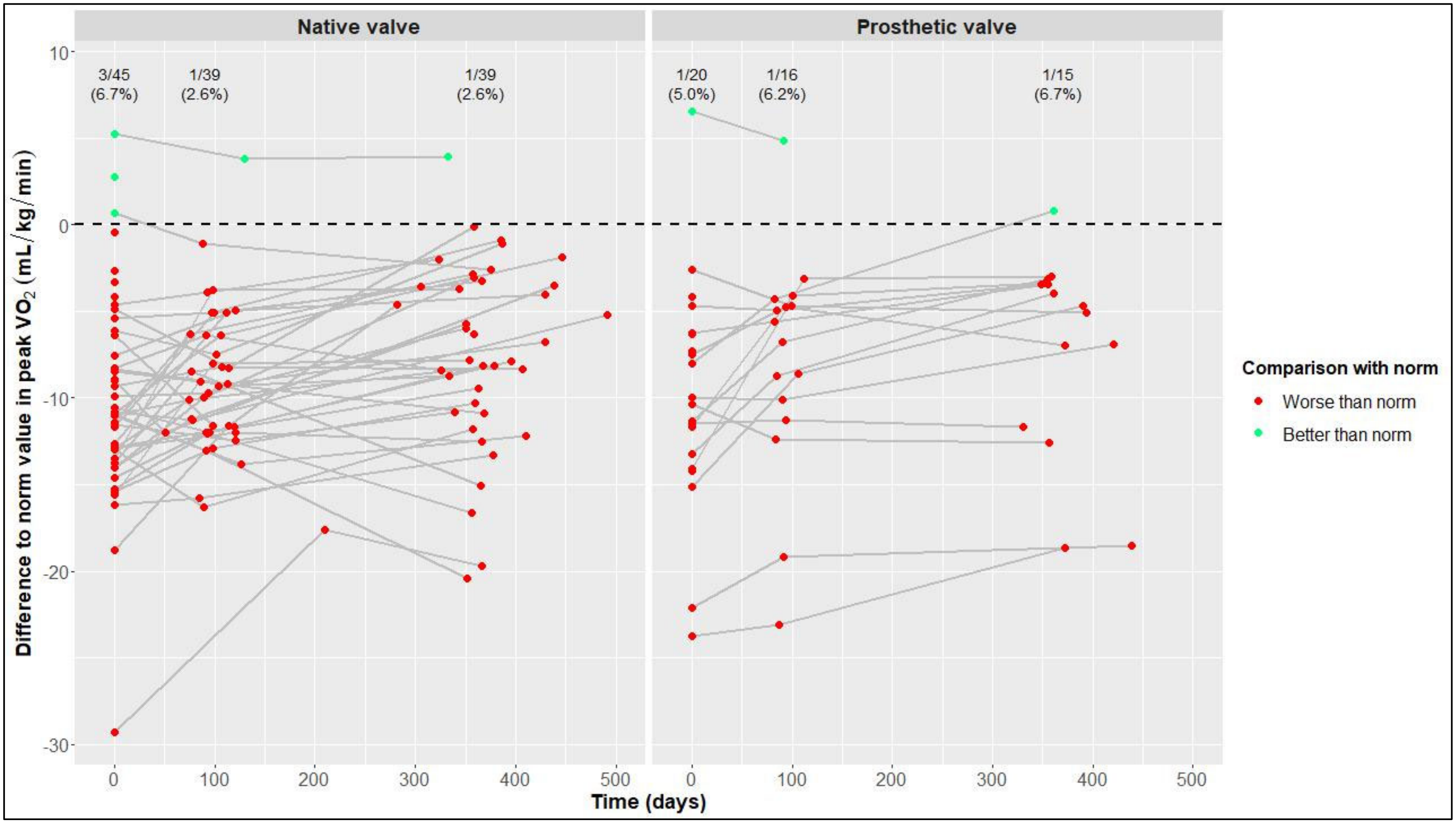

Figure S7. Difference between our values and reference values of work rate at peak oxygen consumption assessed with cardiopulmonary exercise testing (1) in native valve patients vs. prosthetic valve patients; measurements of individual patients are represented by separate dots and connected to show time trend; absolute and relative frequencies of patients reaching reference values are displayed (raw data).

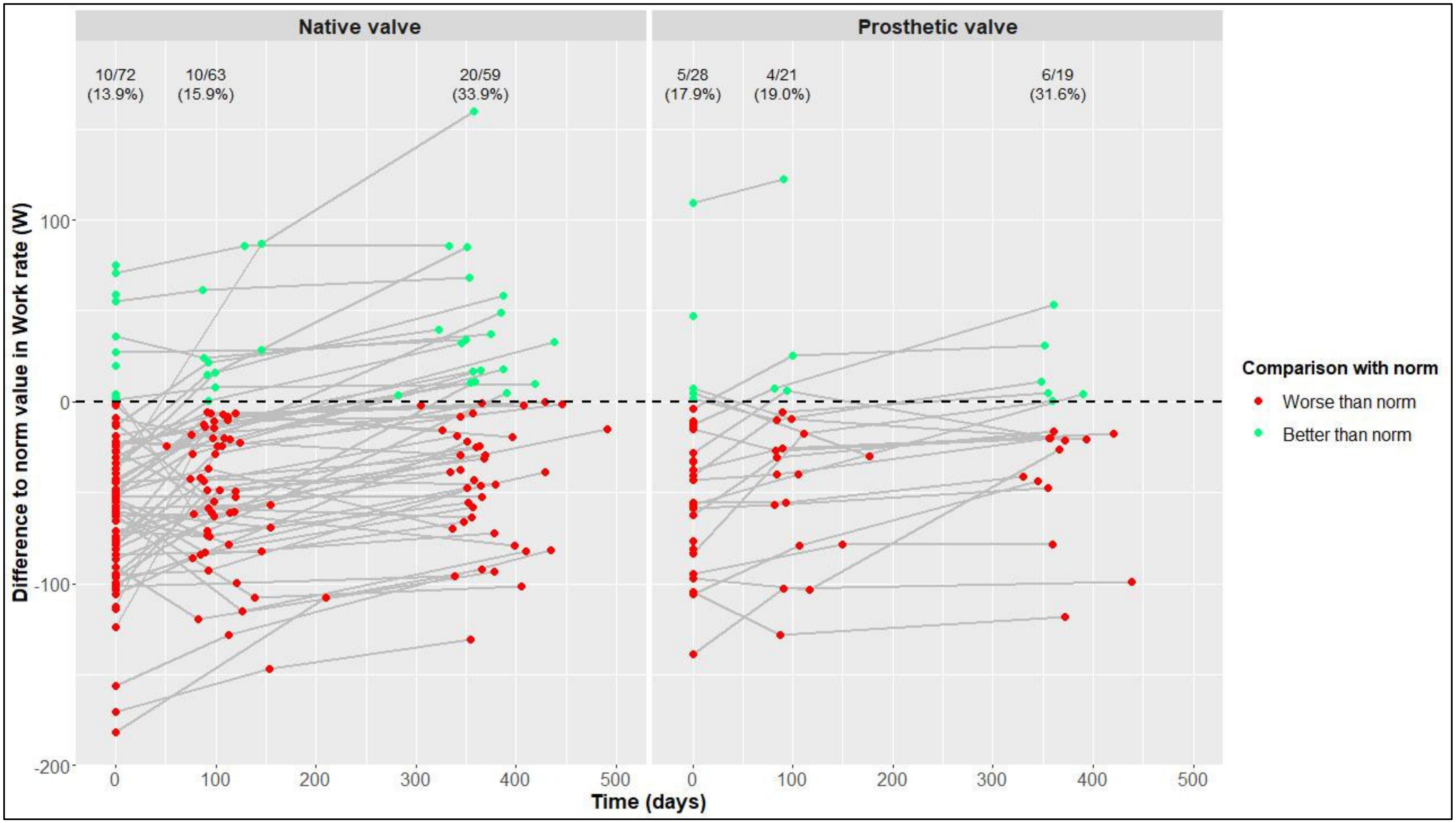

Figure S8. Difference between our values and reference values of self-reported anxiety on Hospital Anxiety and Depression Scale (HADS) (2) in native valve patients vs. prosthetic valve patients; measurements of individual patients are represented by separate dots and connected to show time trend; absolute and relative frequencies of patients reaching reference values are displayed (raw data).

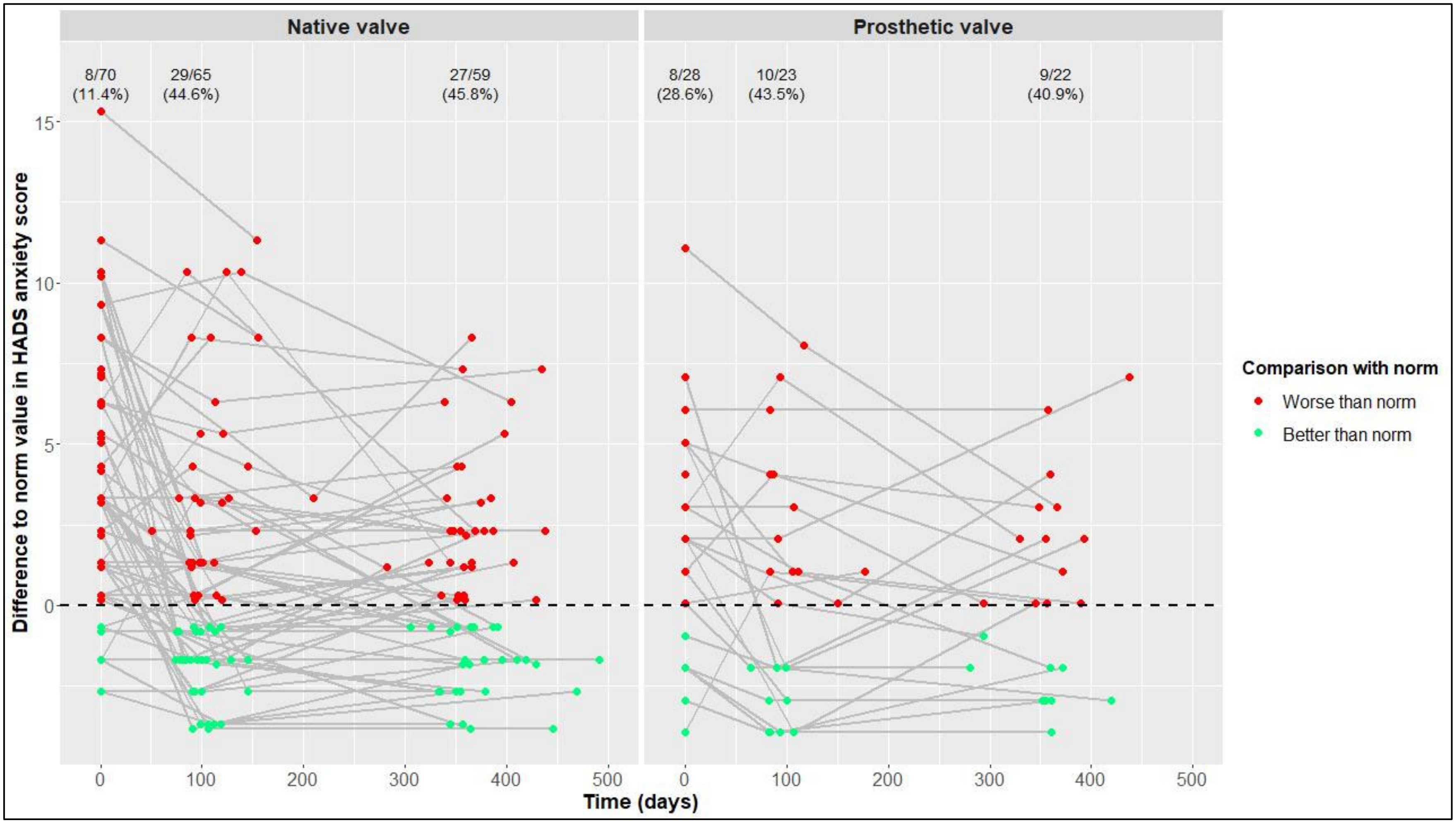

Figure S9. Difference between our values and reference values of self-reported depression on Hospital Anxiety and Depression Scale (HADS) (2) in native valve patients vs. prosthetic valve patients; measurements of individual patients are represented by separate dots and connected to show time trend; absolute and relative frequencies of patients reaching reference values are displayed (raw data).

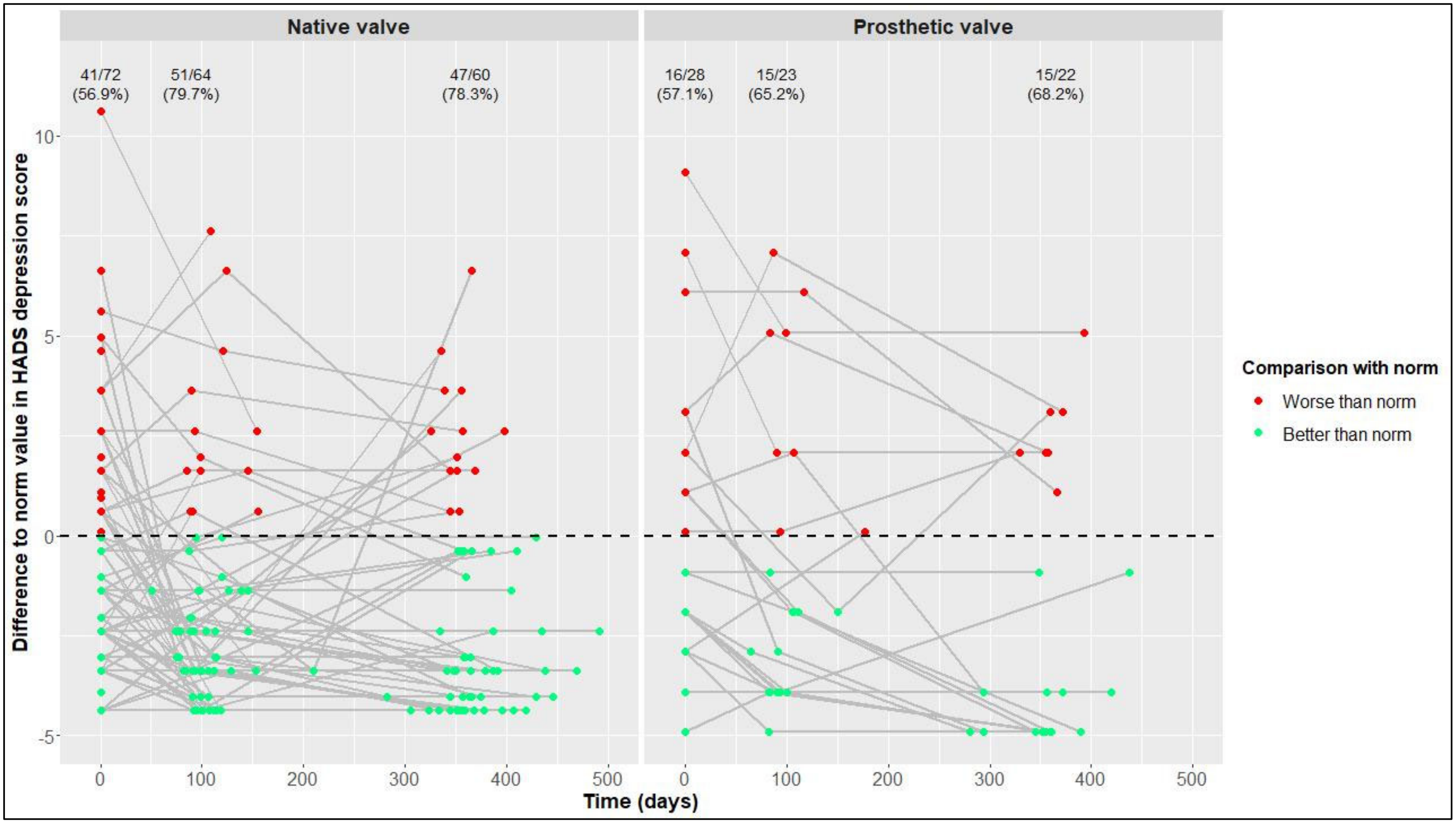

3 References

1. Wasserman K, Hansen JE, Sue DY, Stringer WW, Whipp BJ. *Principles of Exercise Testing and Interpretation: Including Pathophysiology and Clinical Applications*. Philadelphia: Lippincott Williams & Wilkins (2005).

2. Hinz A, Brahler E. Normative Values for the Hospital Anxiety and Depression Scale (Hads) in the General German Population. *J Psychosom Res* (2011) 71(2):74-8. Epub 2011/07/20. doi: 10.1016/j.jpsychores.2011.01.005.
